# Precise Sn-Doping Modulation for Optimizing CdWO_4_ Nanorod Photoluminescence

**DOI:** 10.3390/ijms232315123

**Published:** 2022-12-01

**Authors:** K. Manjunatha, Ming-Kang Ho, Tsu-En Hsu, Hsin-Hao Chiu, Tai-Yue Li, B. Vijaya Kumar, P. Muralidhar Reddy, Ting San Chan, Yu-Hao Wu, Bi-Hsuan Lin, Artashes Karmenyan, Chia-Liang Cheng, Ashish Chhaganlal Gandhi, Sheng Yun Wu

**Affiliations:** 1 Department of Physics, National Dong Hwa University, Hualien 97401, Taiwan; 2 National Synchrotron Radiation Research Center, Hsinchu 30076, Taiwan; 3 Department of Chemistry, University College of Science, Osmania University, Hyderabad 500007, Telangana, India; 4 Department of Material Science and Engineering, National Yang Ming Chiao Tung University, Hsinchu 30010, Taiwan; 5 Department of Electrical Engineering, National Tsing Hua University, Hsinchu 30013, Taiwan

**Keywords:** Sn-doping, photoluminescence, CdWO_4_ rods, Raman spectra, synchrotron-based PXRD

## Abstract

The cadmium tungstate rods have been given much attention due to their potential for usage in numerous luminescent applications. We have prepared single crystalline Sn-doped Cd_1−x_Sn_x_WO_4_ (where x = 0, 1, 3, and 5%) nanorods (NRDs) and characterized them using refined X-ray diffraction and TEM analysis, revealing a monoclinic phase and a crystallite size that decreased from 62 to 38 nm as Sn concentration increased. Precise Sn doping modulation in CdWO_4_ NRDs causes surface recombination of electrons and holes, which causes the PL intensity to decrease as the Sn content rises. The chromaticity diagram shows that an increase in the Sn content caused a change in the emission color from sky blue to light green, which was attributed to the increased defect density. The photoluminescence time decay curve of all samples fit well with double-order exponential decay, and the average decay lifetime was found to be 1.11, 0.93, and 1.16 ns for Cd_1−x_Sn_x_WO_4_, x = 0, 1, and 5%, respectively. This work provides an understanding of the behavior of Sn-doped CdWO_4_ NRDs during electron transitions and the physical nature of emission that could be used in bio-imaging, light sources, displays, and other applications.

## 1. Introduction

Metal tungstates have attracted much attention recently due to their intriguing physical and chemical characteristics and future uses in various sectors such as photochromism, magnetic devices, photonics, photoluminescence, sensors, scintillators, etc. [1,2,3,4]. The tungstate compounds have several advantages, including non-hygroscopicity, thermal and chemical stability, and non-toxicity [5,6]. Self-activating phosphor CdWO_4_, one of the tungstate compounds, can crystallize in either a scheelite tetra agonal structure or a monoclinic wolframite structure. CdWO_4_ is a good candidate host material for phosphors [7]. The intrinsic emission of CdWO_4_ nanorods (NRDs) appears as a broad band in the blue and green regions. This is primarily because of the charge transfer transition between the oxygen ion’s 2p and tungsten ion’s 5d orbitals and self-trapped excitons at the WO_6_^6−^ complex [8]. Because of characteristics such as a high X-ray absorption coefficient, less radiation damage, a high average refractive index, and a broadband gap, the monoclinic structure is a multifunctional material [2,9,10,11]. Oxytungstate with a second-group metal ion is an appealing substance and has drawn tremendous study interest because of its fascinating luminescence and structural characteristics [12,13].

Numerous studies on the luminescence characteristics of doped CdWO_4_ have been published to date [14,15,16,17,18,19,20,21], including those on surface flaws and their effects on the structural and photoluminescence characteristics of Eu^3+^-doped CdWO_4_ nanocrystals [9], photoluminescence studies and a core shell model approach for rare earth doped CdWO_4_ nano phosphors [22], the fluorescence properties for divalent and trivalent europium ions in CdWO_4_ single crystals produced by the Bridgman method [7], the photoluminescence properties of CdWO_4_ doped with Sm^3+^ concentration and annealing effects [23], and photoluminescence characteristics of Ce^3+^ substituted CdWO_4_ nano phosphors [24]. Recently, a variety of Cu- and Bi-doped CdWO_4_ NRDs synthesized by our research team using a hydrothermal approach was successfully used to demonstrate improved photocatalytic performance when exposed to sunlight irradiation [25,26]. As a result, the absorption edge moves into the visible spectrum and captures the photogenerated electrons, reducing the rate of electron-hole recombination. Furthermore, Sn-doping has been shown to improve photoluminescence characteristics compared to the pristine compound [27,28,29,30,31]. To the best of the author’s knowledge, Sn-doped CdWO_4_ has not yet been reported, based on observations of prior studies.

Therefore, in the present study, Sn-doped CdWO_4_ NRDs were prepared using the hydrothermal method. To understand the structural properties and crystalline phase of the samples were studied by using synchrotron-based powder X-ray diffraction (PXRD), microstructural properties of all samples were analyzed by using field-emission scanning electron microscopy (FE-SEM) and field-emission transmission electron microscopy (FE-TEM). Vibrational bands were studied by using Raman spectra, and we have also investigated the effect of Sn on photoluminescence properties of CdWO_4_ NRDs at low-temperature and at room temperature. Our in-depth material characterization correlates microstructural, structural, Raman, and photoluminescence properties, confirming Sn doping into the CdWO_4_.

## 2. Results and Discussion

**Figure 1:**
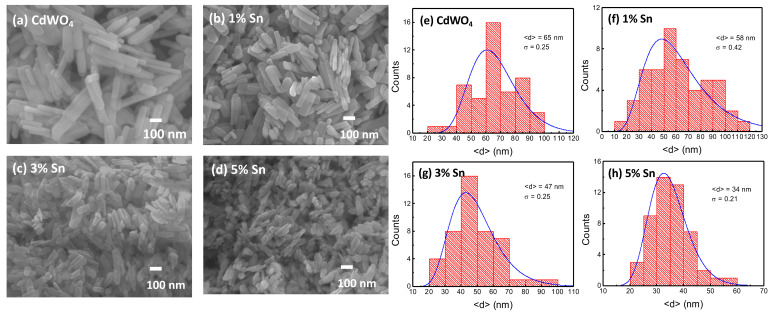
(**a**–**d**) FE-SEM images of CdWO_4_ and 1, 3, 5% Sn-doped CdWO_4_ NRDs. (**e**–**h**) The histogram distribution in the diameter of Cd_1−x_Sn_x_WO_4_ (x = 0, 1, 3, and 5%) NRDs.

**Morphological characterization.** FE-SEM was used to examine the morphologies of pure and Sn-doped CdWO_4_; the high and low magnification images are shown in Figure 1a–d and Figure S1 (Supporting Information), respectively. The FE-SEM images of CdWO_4_ and Sn-doped CdWO_4_ clearly show the rod-like morphology, which is consistent with the literature [18]. The mean diameter $\langle d_{\mathrm{SEM}}\rangle$ of rods is obtained by fitting a log-normal distribution function:

(1) $$f\left(d\right)=\frac{1}{d\sigma \sqrt{2\pi }}\exp\left[-\frac{{\left(\mathrm{lnd}-\langle d_{\mathrm{SEM}}\rangle \right)}^{2}}{2\sigma^{2}}\right]$$

to the histogram obtained from the FE-SEM images, as shown in Figure 1e–h, where the value of σ represents a standard deviation of the fitted function [18]. The nanorod length histogram distributions were also shown in Figure S2 (Supporting Information). All obtained fitted parameters were summarized in Table S1 (Supporting Information). An increase in Sn content from 0 to 5% causes an increase in aggregation, a decrease in diameter from 65 to 34 nm, and a decrease in length from 317 to 115 nm, resulting in an astonishing ~31% reduction in the aspect ratio, as shown in Figure S3 (Supporting Information). According to the above analysis of the FE-SEM images, the reduction in CdWO_4_ crystals is caused by Sn doping. The above findings are in line with studies that have been published on Cu-, Fe-, La-, and Eu-doped CdWO_4_ and Cd-doped ZnWO_4_ [1,28,32,33,34,35,36]. Therefore, doping changes the CdWO_4_ lattice structure regardless of the type of dopant used, and it may even prevent crystal formation by changing the surface charge [37]. According to the SEM pictures, the mean size reduction at the increased Sn content causes high surface energy, which causes aggregation (Figure S1, Supporting Information). These results show that aggregation occurred in the reaction mixture and that Sn content can effectively control it. Figure S4a–d (Supporting Information) shows the EDS analysis of Sn-free and Sn-doped CdWO_4_, respectively. According to the EDS analysis, there are no impurities in the synthesized samples. Furthermore, the energy dispersive absorption X-ray spectroscopy confirmed the previously reported uniform distribution of all constituent elements [26].

**Figure 2:**
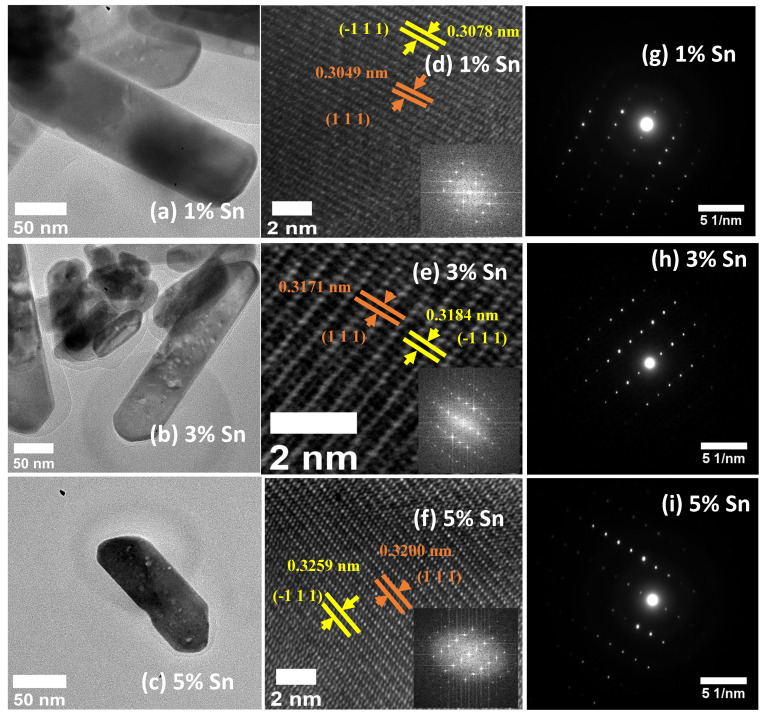
(**a**–**c**) TEM images, (**d**–**f**) high-resolution TEM images, and (**g**–**i**) SAED pattern of 1, 3, and 5% Sn-doped CdWO_4_ NRDs (top to bottom).

Figure 2a–c depict the TEM images of 1% Sn, 3% Sn, and 5% Sn doping CdWO_4_ NRDs, revealing a decreased diameter of the nanorods with increased Sn concentration. Figure 2d shows the high-resolution TEM image of pure 1% Sn-doped CdWO_4_ NRDs, yielding lattice spacing of 0.3078 and 0.3049 nm for (−1 1 1) and (1 1 1) planes, respectively. Similarly, 3% and 5% Sn-doped CdWO_4_ NRDs yield the lattice spacing [0.3184, 0.3171 nm] and [0.3259, 0.3200 nm], corresponding to the [(−1 1 1), (1 1 1)] planes, respectively (Figure 2e,f) [38]. The mean value of d-spacing obtained from high-resolution images is shown in Figure S5 (Supporting Information). The obtained lattice spacing values corresponding to the (1 1 1) plane well agrees with the reported value elsewhere [38]. The 1, 3, and 5% Sn-doped CdWO_4_ oriented along the [0 1 −1] zone axis and the SAED patterns of 1%, 3%, and 5% Sn-doped CdWO_4_ NRDs confirm the monoclinic structure (Figure 2g–i). The Fourier transform generated from HRTEM images (inset of Figure 2d–f) are in agreement with the SAED pattern acquired from a single NRD. The SAED patterns of 1%, 3%, and 5% Sn-doped CdWO_4_ NRDs confirm the monoclinic structure (Figure 2g–i). The TEM analysis results agree with the XRD results and confirm that the 1%, 3%, and 5% Sn-doped CdWO_4_ NRDs are purely monoclinic crystal structures.

**Figure 3:**
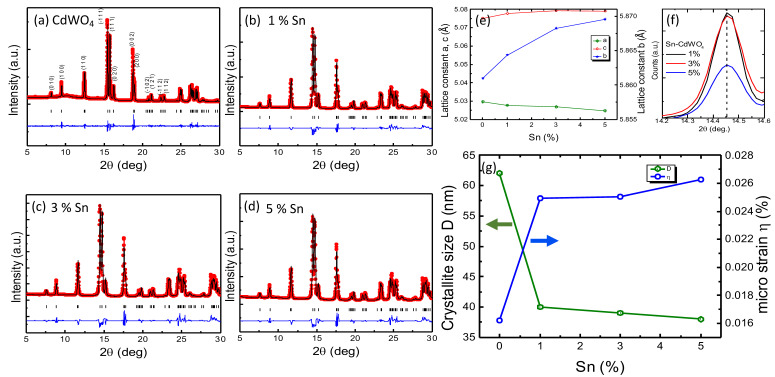
(**a**–**d**) The refined PXRD patterns of Cd_1−x_Sn_x_O_4_ with x = 0 (CdWO_4_), 1, 3, and 5%. (**e**) Variation of lattice constants with Sn concentrations. (**f**) Diffraction peak (−1 1 1) from all the samples where the vertical dashed line marks the peak center of the CdWO_4_ nanorod. (**g**) Variation of crystallite size and microstrain with Sn concentrations.

**Structural Characterizations.** The refined PXRD patterns of Cd_1−x_Sn_x_O_4_ with x = 0 (CdWO_4_), 1, 3, and 5% are shown in Figure 3a–d. The monoclinic structure of the JCPDS-14-0676 file [39], which has a space group of *P2/C*, is matched by the diffraction peaks seen in the XRD patterns, confirming the single phase. The Rietveld refinement approach was used to confirm the phase purity firmly. The refined diffraction patterns are displayed in Figure 3a–d, where there is a slight variation, nearly zero, between the calculated (red) and absolute intensities (black) [40]. The dopant Sn ions may have been successfully incorporated into the lattice because there are no additional peaks in the pattern and any other impurity phases [41]. Table S2 (Supporting Information) contains an occupancy of Sn-doped CdWO_4_ derived by Rietveld refinement. Using the Fullprof software, the lattice parameters and volumes have been determined and summarized in Table 1. Because of the difference in ionic radius between Sn^2+^ (0.69 Å) and Cd^2+^ ions (0.97 Å), the lattice parameters varied a small amount as Sn concentration increased (Figure 3e) [42]. Figure 3f shows a small clear shift in diffraction peaks (−1 1 1) with increasing Sn concentrations. This shifting could be caused by differences in the nuclear radius of the Sn and Cd atoms, which results in a small variation in lattice constant value, as shown in Table 1. The Sm^3+^-doped CdWO_4_ has exhibited similar behavior, according to recent reports [23]. Unit cell volume decreases with increases in Sn^2+^ concentration due to the ionic radius of Cd^2+^ ions (0.97 Å) and ions greater than the Sn^2+^ (0.69 Å) ions, as can be seen in Table 1. Figure S6a–d (Supporting Information) displays the fitting of the Gaussian model to the (−1 1 1) diffraction peak [43]. It can be seen that the entire FWHM value of the fitting peaks increased with rising Sn concentrations. The Williamson–Hall (WH) plot method (Equation (2)) was used to calculate the crystallite size and microstrain [44,45]:

(2) $$\varepsilon =\varepsilon_{\mathrm{size}}+\varepsilon_{\mathrm{strain}}=\frac{1}{\cos\theta }\left(\frac{k\lambda }{\langle d_{\mathrm{XRD}}\rangle }+4\eta \sin\theta \right)$$

where k is a constant, β_(hkl)_ is the full width half maximum (FWHM), λ is the wavelength of the incident X-ray, and ε represents FWHM. The WH plot is displayed in Figure S7 (Supporting Information), and the obtained mean crystallite size is shown in Table 1. The calculated crystallite size from the WH method agrees with the reported value for Cu-CdWO_4_ NRDs [32]. As shown in Figure 3g, the observed microstrain (%) value increases with increasing Sn content, indicating an increase in [MO^6^] (M = Cd and Sn) cluster deformation brought on by the incorporation of Sn^2+^ into the CdWO_4_ [34]. By increasing Sn concentration the diffraction peak may broaden, which may be associated with a reduction in crystallite size and an increase in strain, showing that the Sn ions have successfully entered the CdWO_4_ lattice [46]. This outcome makes it evident that the dopant Sn ions were successfully incorporated into the lattice.

**Figure 4:**
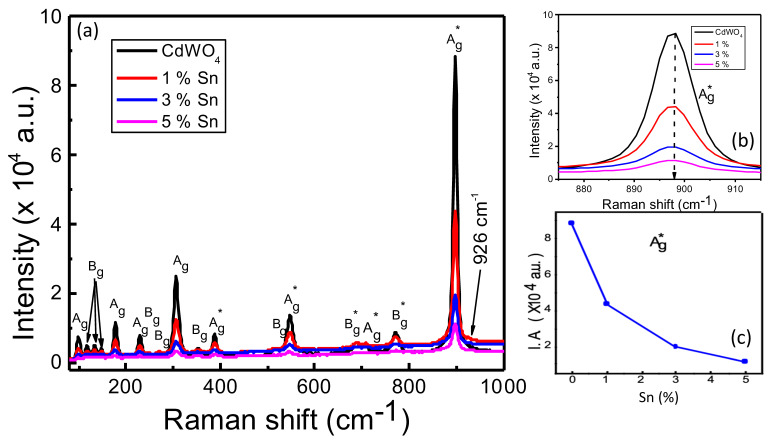
(**a**) Raman spectra of Cd_1−x_Sn_x_WO_4_ (x = 0, 1, 3, and 5%) NRDs (The asterisks (*) indicate the internal stretching modes). (**b**) A plot of the vibrational band observed at 898 cm^−1^ from 0 to 5% Sn-doped CdWO_4_ NRDs. (**c**) The integrated intensity vs. Sn doping concentrations.

**Raman scattering.** Group theory simulations show that tungstates with monoclinic or triclinic structures exhibit 36 distinct Raman vibrational modes (8A_g_ + 10B_g_ + 8A_u_ + 10B_u_), 18 of which are anticipated to be active modes (8A_g_ + 10B_g_). In this study, Raman spectra were collected at wavenumber ranging from 80 to 1000 cm^−1^. The Raman spectra of Cd_1−x_Sn_x_WO_4_ NRDs (x = 0, 1, 3, and 5%) are displayed in Figure 4a. All samples had a vital vibration peak at 898 cm^−1^. Several weak vibrations were at 772, 707, 687, 548, 516, 387, 352, 307, 269, 248 230, 178, 148, 134, 117, and 99 cm^−1^ [32,47]. In this study, 17 active Raman modes were observed, which is completely in line with the findings reported by Ross-Medgaarden and Wachs [36]. According to previously reported works, one more Raman mode will be observed at 77cm^−1^. Thus, in the current work, we observed only 17 active Raman modes because we studied the Raman spectra from 80 to 1000 cm^−1^, confirming the monoclinic structure in our samples [32,36]. In general, external (600 cm^−1^) and internal (>600 cm^−1^) vibrational modes found in the Raman spectra of tungstates may be divided into two groups, according to the reported literature [32,48]. The vibrational bands in the 150–300 cm^−1^ region were attributed to the Cd–O stretching modes. The symmetric stretching of [CdO_6_] octahedra is responsible for the vibrational band at 307 cm^−1^. The vibration bands in the 500–600 cm^−1^ region are attributed to symmetric W–O–W stretching modes. The vibrational bands 687 and 772 cm^−1^ modes involve [WO_6_] octahedra motions against the Cd^2+^. Normal WO vibration of [WO_6_] octahedra corresponds to the 387 and 898 cm^−1^ modes. The line width broadening concerning Sn content is observed in all the peaks. A Raman shift of ~2 cm^−1^ was observed when Sn doping concentration increased from 0 to 5% (230 to 228 cm^−1^ and 898 to 896 cm^−1^ vibration modes, as shown in Table S3, Supporting Information). With increasing Sn^2+^ concentration, a Raman shift was observed, indicating lattice distortion in the Sn^2+^-doped CdWO_4_ nanorod [49]. The most intense Raman band observed around A_g_*~898 cm^−1^ in undoped and Sn-doped samples characterized by the WO_6_ symmetric stretching vibration (Figure 4b). A similar Raman band has also been reported around 891 cm^−1^ from NiWO_4_ nanopowders [50]. The integrated peak intensity decreases with the increase in Sn concentration, as shown in Figure 4c. It should also be noted that a new Raman band at 926 cm^−1^ was discovered from a 1% Sn sample. The intensity of the new peak decreases as Sn concentrations increase. A similar Raman band was also reported around 932 cm^−1^ from 1% Sn-doped CdWO_4_ NRDs ascribed to surface disorder layers [32]. As a result, there is evidence that the Sn dopant caused structural inhomogeneity.

**Figure 5:**
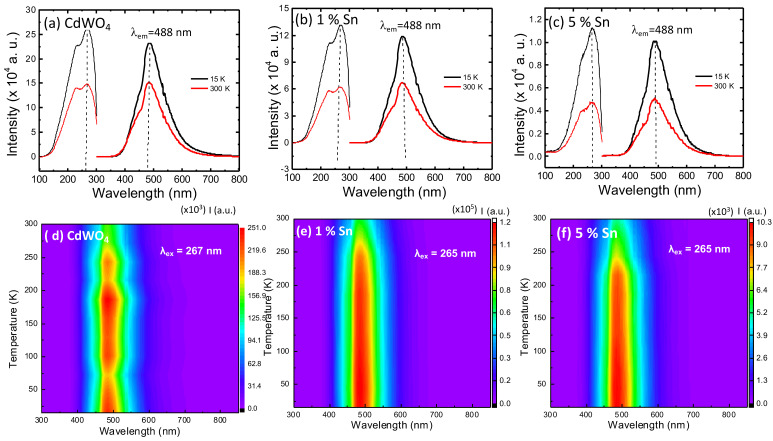
(**a**–**c**) The excitation and emission spectrum obtained at 15 K (black) and 300 K (red) (**d**–**f**), a 2D plot of the emission spectrum, where the vertical axis represents temperature varying from 15 to 300 K.

**Figure 6:**
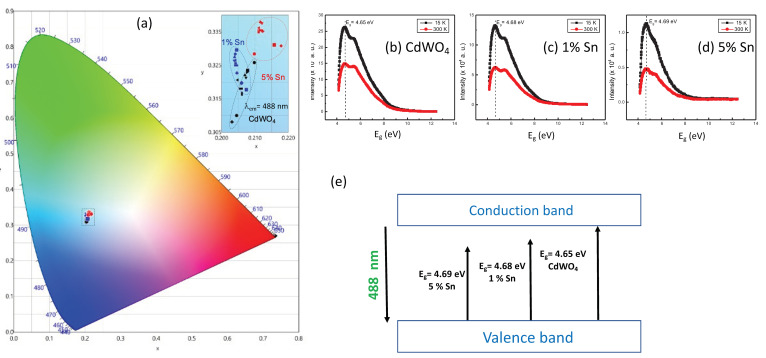
(**a**) CIE 1931 color space chromaticity diagram in the (X, Y) coordinate system shows a shift in the emission color from light blue to sky blue of the PL emission with increased Sn-content. The plot on the right-hand side shows the magnified rectangular area. (**b**–**d**) Temperature-dependent photoluminescence spectra of undoped and Sn-doped CdWO_4_ for the most intense peak at the direct bandgap at room temperature and lower temperatures. (**e**) Schematic diagram of photoluminescence mechanism of Cd_1−x_Sn_x_WO_4_ (x = 0, 1 and 5%) NRDs.

**Photoluminescence studies.** The emission spectrum of Sn-free CdWO_4_ and Sn-doped CdWO_4_ NRDs were obtained at 300 K (red) and 15 K (black), respectively, as shown in Figure 5a–c. The A11→T31 transitions in the [WO_6_] complex are the only ones responsible for the PL emission in CdWO_4_ [18]. The excitation spectra of Sn-doped CdWO_4_ consist of a broad band in the wavelength ranges of 100 to 315 nm, with the most significant intensities at approximately 267, 265, and 265 nm for 0, 1, and 5%, respectively, at 15 K. This broadband can be attributed to the overlap of ligand to metal charge transfer transitions between oxygen to Sn^2+^ and the filled 2p orbital of oxygen and the empty 5d orbital of W^6+^ within the WO_6_^6−^ complex [1,46,51]. The most vigorous UV emission (267 nm) was found at x = 0 and gradually weakened with Sn content doping. Regarding the visible region’s emission, Sn-doped CdWO_4_ nanocrystals showed the weakest emission. One crucial observation is that the UV and visible emissions’ relative PL intensities decreased with the increasing Sn dopant. This leads us to the conclusion that the electron relaxation time of the conduction band is prolonged due to the charge transfer between the conduction band and the impurity levels [52]. CdWO_4_ NRDs are excited by 267 nm UV light, which results in just one strong blue-green emission spectrum in the range of 350 to 700 nm, with a peak centered at 484 nm near the values that have been reported [18]. The charge transfer transition inside the WO_6_^6−^ complex is also responsible for the broad band of intense wavelengths that was also seen toward the higher wavelength regions in the ranges between 330 and 600 nm, in addition to these peaks around 484 nm attributed to (6H_5/2_-4F_7/2_), which agree with the reported values [32,53,54]. The asymmetry is due to the existence of energetically distinct O(1) and O(2) sites such that the decay process of ${\mathrm{WO}}_{6}^{6-}$ groups can occur via two slightly different electronic transitions [32]. The emission peak profile did not significantly change as Sn concentrations varied; but, nevertheless, Sn concentrations considerably impacted photoluminescence intensity. There is an efficient energy transfer from the host lattice to dopant Sn^2+^ because there is significant broadband owing to O^2^-W^6+^/Sn^2+^ as contrasting to the f-f transitions of Sn [51]. Additionally, it was found that the emission intensity reduced as temperature increased from 15 to 300 K, possibly because rising temperatures increase the elimination of luminescence. Photoluminescence spectra also show that broadening of emission and excitation peaks decreases the temperature from 300 to 15 K. There was no shift in emission or excitation peak with increasing temperature. After doping Sn, their interactions grow, eventually leading to cross-relaxation, which causes the emission intensity to drop when the mean distance between them is less than a critical value. More photogenerated electrons and holes can participate in the oxidation and reduction reactions when the PL intensity is lower. Similar characteristics preserved in the Sn-doped samples with a more pronounced quench indicate a decrease in electron and hole recombination rate with increasing Sn content. With Sn doping, the photocatalytic performance can be enhanced from an application standpoint. The temperature-dependent PL measurement was carried out to look at the optical characteristics of Sn-doped CdWO_4_ below room temperature. The 2D mapping of the temperature-dependent PL spectra with intensity is shown in Figure 5d–f. There are emission peaks at low temperatures. Internal quantum efficiency (IQE), which is measured as the ratio of integrated PL intensity between 15 and 300 K and assumes an internal efficiency of 100% at a low temperature of 15 K, was found to be 64.7%, 55.9%, and 48.9% for 0, 1%, and 5% Sn, respectively. This shows a relative decrease in the IQE of 5% Sn-doped CdWO_4_ NRDs of about 49% compared to pure CdWO_4_ NRDs. The PL intensity, however, monotonically decreases in Sn CdWO_4_ NRDs. Sn doping in CdWO_4_ causes surface recombination of electrons and holes, which causes the PL intensity to decrease as Sn content rises. The recombination and transfer of photogenerated electron–hole pairs also can be caused by a decrease in PL intensity as Sn content increases. It is generally acknowledged that, from an application standpoint, Sn-doped CdWO_4_ can be stronger than the photocatalytic performance [32]. The CIE 1931 chromaticity coordinates (X, Y) were next acquired to assess the performance of the Sn-doped CdWO_4_ NRDs on color luminescent emission. Figure 6a depicts the resulting chromaticity diagram. It demonstrates that increased Sn content caused a change in the emission color from sky blue to light green, which was attributed to the increased defect density.

The temperature-dependent photoluminescence excitation (PLE) spectra of undoped and Sn-doped CdWO_4_ for the most intense peak was at the direct bandgap at 300 and 15 K temperatures, as shown in Figure 6b–d [55,56]. For CdWO_4_, the maximum intensity of the PLE band is located at 4.65 eV. A similar profile was obtained for the 5% Sn-doped CdWO_4_ sample with a band located at 4.69 eV. The temperature-dependent photoluminescence emission spectra of undoped and Sn-doped CdWO_4_ for the emission peak are located at 2.56 eV (484 nm), as shown in Figure S8 (Supporting Information). Electron-phonon coupling could explain the temperature dependence of emission peaks [57]. Due to the thermal quenching caused by nonradiative recombination, the emission intensity drops as the temperature rises. Figure 6e reveals the photoluminescence mechanism of Cd_1−x_Sn_x_WO_4_ NRDs with various Sn concentrations. At low temperatures and room temperature, the primary emission peak for both doped and undoped samples was located at approximately 4.65 eV for CdWO_4_, which corresponds to the direct energy gap. After doping the 5% Sn to the CdWO_4_, the energy gap increases to 4.69 eV. The band gap obtained from the pristine CdWO_4_ NRDs matched what was reported elsewhere [57,58]. It was found that increased Sn doping caused an increase in energy gap values, which indicates that the doping caused the absorption edge to shift toward higher energies. This increase can be explained by the Bureshtain–Moss Shift phenomenon, in which the levels close to the conduction beam are dense with electrons which requires more energy for the electrons to travel, giving the impression that the energy gap is expanding. Similar behavior was also observed in the reported Sn-doped cadmium oxide [59].

**Figure 7:**
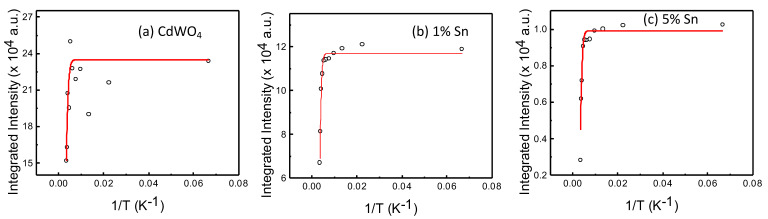
(**a**–**c**) The temperature-dependent integrated PL intensity of Cd_1−x_Sn_x_WO_4_ (x = 0, 1, and 5%) NRDs.

The temperature-dependent integrated PL intensity has been studied at temperatures ranging from 15 to 300 K to learn more about the origin of PL emission. Figure 7a–c depicts the variance in Integrated PL intensity as a function of inverse temperature. The temperature dependence of PL intensity has been significantly changed by Sn-doping. A similar response was observed in Cu-doped BaWO_4,_ NiWO_4_, and CdWO_4_, allocated to dopant-induced intermediate energy levels [27,28,32]. For further brief analysis, the integrated PL intensity as a function of inverse temperature for CdWO_4_ (0% Sn) has been fitted using the Arrhenius equation, as shown below [60]:

(3) $$I\left(T\right)=\frac{I_{0}}{\left[1+A\exp\left(-E_{1}/k_{B}T\right)\right]}$$

where $I_{0}$ is the integrated PL intensity at temperature 15 K, *I*(*T*) is the integrated PL intensity at temperature T, and $k_{B}$ is Boltzmann’s constant. We propose that the quenching mechanism of PL intensities for CdWO_4_ NRDs is connected to two non-radiative recombination processes based on fitting data. So, the experimental data (Figure 7b,c) is fit by using a modified Arrhenius equation, as shown below:

(4) $$I\left(T\right)=\frac{I_{0}}{\left[1+A\exp\left(-E_{1}/k_{B}T\right)+B\exp\left(-E_{2}/k_{B}T\right)\right]}$$

where $E_{1}$ and $E_{2}$ implies the activation energies of the non−radiative recombination for thermal quenching of PL intensities at a higher and lower temperature. A and B are the constants that represent the ratio of $\tau_{R}$ (radiative lifetime of carriers) and $\tau_{0}$ (non-radiative lifetime of carriers). Table S4 provides an overview of the best-fit parameters (Supporting Information). From the best fitting of the data using the Arrhenius equation, activation energy $E_{1}$ for PL quenching is found to be 145.72 meV for CdWO_4_. $E_{1}$ emerged as a potential barrier between the localized potential minima and the non-radiative centers in the numerous quantum wells in this case [32]. As observed in the best-fitted modified Arrhenius equation, a decrease in the fitted value of $E_{1}$ from 192.73 to 108.9 meV with Sn concentration increases, ranging from 1 to 5%, suggesting that carriers can easily escape from confined states at higher temperatures. The constant A increases from 154 to 1321, with an increase in Sn from 0 to 5% due to a decrease in $\tau_{0}$. The density of non-radiative recombination centers decreases as Sn content increases, which causes a decrease in A, consistent with previous earlier reports [32,60]. The low $E_{2}$ and B values obtained for the 1 and 5% Sn-doped CdWO_4_ NRDs consist of quenching at low temperatures.

**Figure 8:**
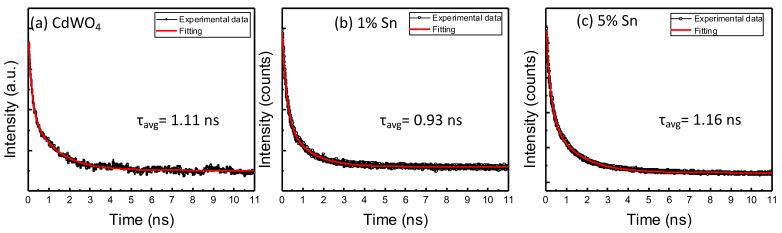
(**a**–**c**) Photoluminescence decay curves of Cd_1−x_Sn_x_WO_4_ (x = 0, 1, and 5%) NRDs.

Figure 8a–c shows the photoluminescence time decay curves of Cd_1−x_Sn_x_WO_4_ NRDs (x = 0, 1, and 5%). All the curves fit well with double-order exponential decay, as shown below [61]:

(5) I(t)=I0+A1exp(−tτ1)+A2exp(−tτ2)

where *I* represents the photoluminescence intensity, *I*_0_ is the photoluminescence intensity at 0, and *A*_1_ and *A*_2_ are constants. *τ*_1_ and *τ*_2_ represent the decay times for the exponential components. Average decay lifetimes can be calculated as follows [61]:

(6) $$I\left(t\right)=(A_{1}\tau_{1}^{2}+A_{2}\tau_{2}^{2})/\left(A_{1}\tau_{1}+A_{2}\tau_{2}\right)$$

Using Equation (6), it is possible to calculate the decay times of Cd_1−x_Sn_x_WO_4_ NRDs (x = 0, 1, and 5%); they were found to be 1.11, 0.93, and 1.16 ns, respectively. This result shows lifetimes varied with increasing Sn^2+^ concentration. It can strongly prove the energy transfer process existing from Sn^2+^ ions [61].

## 3. Methods and Materials

**Scheme 1:**
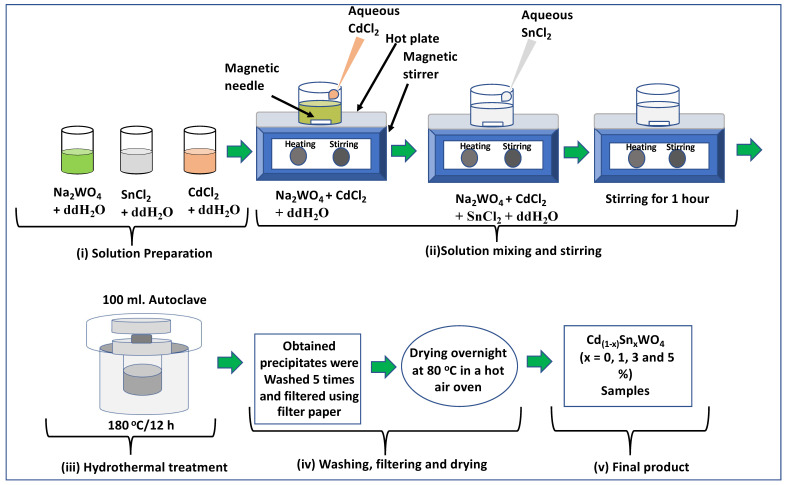
Schematic representation of solution preparation, filtering and drying of Cd_1−x_Sn_x_WO_4_ (x = 0, 1, 3, and 5%) samples using in situ hydrothermal methods.

The Cd_1−x_Sn_x_WO_4_ (x = 0, 1, 3, and 5 %) NRDs were prepared using in situ hydrothermal methods [26]. Scheme 1 shows the schematic representation of the synthesis process of Cd_1−x_Sn_x_WO_4_ (x = 0, 1, 3, and 5%) samples using in situ hydrothermal methods. In this method, the stoichiometric quantity of tungstic acid sodium salt dihydrate (Na_2_WO_4_·2H_2_O), tin chloride dihydrate (SnCl_2_·2H_2_O), and cadmium chloride hydrate (CdCl_2_·H_2_O) were dissolved separately in ddH_2_O (double-distilled water). The tungstic acid sodium salt dihydrate solution was then mixed with cadmium chloride hydrate, followed by the dropwise addition of desired tin chloride dihydrate while constantly stirring. The resulting solution, which had different concentrations of Sn, was stirred continuously for an hour before being transferred individually to a 100 mL Teflon-lined stainless-steel autoclave, which made up around 80% of the total capacity. Without shaking or stirring during the heating period, all of the autoclaves with various Sn content solutions were kept at 180 °C for 12 h at once, after which they were allowed to cool naturally to room temperature. The precipitates were collected, filtered, and washed five times with ddH_2_O before being dried at 80 °C overnight in a hot air oven. At first look, the samples’ peculiar color suggested that an increase in the Sn content may have impacted the structural and optical characteristics of CdWO_4_ [62]. Keep in mind that both before and after the reaction, the pH level is near 7. Samples were analyzed morphologically using field-emission scanning electron microscopy (FESEM, JEOL JSM-7000F). TEM characterization was carried out through field-emission transmission electron microscopy (FE-TEM, JEM-F200). The samples were structurally characterized using PXRD at the National Synchrotron Radiation Research Center (NSRRC) Taiwan light source (TLS) 01C2 beamline in Hsinchu, Taiwan (λ = 0.77491 Å). Raman spectra of all the samples were studied using a micro-Raman spectrometer coupled with a microscope (Leica, Wetzlar, Germany) and a 532 nm wavelength laser. The TLS 03A1 beamline of the NSRRC in Taiwan was used to perform the temperature-dependent PL observations. Two-photon fluorescence lifetime imaging (TP-FLIM) has been used to visualize Sn-doped CdWO_4_ NRDs. This measurement was performed with the objective UPlanFLN 40/0.75 (Olympus, Shinjuku, Tokyo, Japan). TP-FLIM measurements and the lifetime imaging of Sn-doped CdWO_4_ NRDs were performed using the excitation of a Ti-sapphire laser (Chameleon Ultra-II; pulse duration 140 fs; repetition rate 80 MHz). The wavelength of excitation used was 760 nm. The excitation light power was controlled by a neutral density filter and was measured before the microscope objective from 15 to 35 mW. The emission was collected in the range of 450 to 650 nm with a single photon counting system Pico Harp 300 (Pico Quant, Germany) and a thermoelectrically cooled PMT conjugated with microscope Olympus IX 71. Fluorescence lifetime images of Sn-doped CdWO_4_ NRDs were obtained and analyzed using the SymPho Time software package, Version 5.2.4.0 (Pico Quant, Germany).

## 4. Conclusions

In this work, the monoclinic structure of Cd_1−x_Sn_x_WO_4_ (x = 0, 1, 3, and 5%) NRDs has been synthesized using in situ hydrothermal methods. The FE-SEM and TEM images of pure CdWO_4_ and Sn-doped CdWO_4_ clearly show the nanorod-like morphology. An increase in Sn content from 0 to 5% causes an increase in aggregation, a decrease in diameter from 65 to 34 nm, and a decrease in length from 317 to 115 nm, resulting in an astonishing ~31% reduction in aspect ratio. According to the EDS analysis, there are no impurities in the synthesized samples. SAED patterns confirm the formation of a monoclinic structure. The Rietveld refined XRD patterns demonstrate the single phase without showing any impurities. Increasing Sn concentration may broaden the diffraction peak, associated with a reduction in crystallite size and increased strain. A Raman shift was observed with increased Sn^2+^ concentration, indicating lattice distortion in the Sn^2+^-doped CdWO_4_ NRDs. The emission peak profile did not change significantly as Sn concentrations changed; however, Sn concentrations greatly impacted photoluminescence intensity. The chromaticity diagram shows that an increase in the Sn content caused a change in the emission color from sky blue to light green, which was attributed to the increased defect density. The intensity of the emission peak decreases with increasing temperature due to thermal quenching by nonradiative recombination. The average lifetime of Sn^2+^-doped CdWO_4_ NRDs was obtained from the fitted photoluminescence time decay curves and confirmed that the average lifetimes varied with increasing Sn^2+^ concentration. It can strongly prove the energy transfer process existing between Sn^2+^ and Cd^2+^ ions.

## Figures and Tables

**Table 1:** Summary of the crystallite sizes, lattice parameters, microstrains, and volume obtained from PXRD of Sn-doped CdWO_4_ NRDs.

Sn-Doping	Crystallite Size (D) nm	Lattice Parameters (Å)			η (%)	Volume (Å^3^)
		*a*	*b*	*c*		
0	62	5.0298	5.8610	5.0753	0.01619	149.570
1%	40	5.0279	5.8644	5.0778	0.02495	149.673
3%	39	5.0270	5.8683	5.0794	0.02506	149.786
5%	38	5.0249	5.8696	5.0792	0.02624	149.812

## Supplementary Materials

The following supporting information can be downloaded at: https://www.mdpi.com/article/10.3390/ijms232315123/s1.
