# Supplementary material for: Precise Sn-Doping Modulation for Optimizing CdWO4 Nanorod Photoluminescence
Source: Int J Mol Sci. 2022 Dec 1;23(23):15123. doi: 10.3390/ijms232315123 (PMC9736181; doi:10.3390/ijms232315123)
Supplement: Supplementary file 1 [file ijms-23-15123-s001.zip › ijms-2045139-supplementary.pdf]

Supporting Information

Precise Sn-doping Modulation for Optimizing $CdWO_4$ Nanorods Photoluminescence

K. Manjunatha,[a] Ming-Kang Ho,[a] Tsu-En Hsu,[a] Hsin-Hao Chiu,[a] Tai-Yue Li,[a,b] B. Vijaya Kumar,[c] P. Muralidhar Reddy,[c] Ting San Chan,[b] Yu-Hao Wu,[b,d] Bi-Hsuan Lin,[b] Artashes Karmenyan,[a] Chia-Liang Cheng,[a] Ashish Chhaganlal Gandhi,[a,e] and Sheng Yun Wu[a,*]

[a] Department of Physics, National Dong Hwa University, Hualien 97401, Taiwan

[b] National Synchrotron Radiation Research Center, Hsinchu 30076, Taiwan

[c] Department of Chemistry, University College of Science, Osmania University, Hyderabad 500007, Telangana, India

[d] Department of Material Science and Engineering, National Yang Ming Chiao Tung University, Hsinchu 30010, Taiwan

[e] Department of Electrical Engineering, National Tsing Hua University, Hsinchu 30013, Taiwan

* Corresponding authors: sywu@mail.ndhu.edu.tw (SYW)

Figure S1 (a)-(d) shows the low magnification FE-SEM images of $Cd_{1-x}Sn_xWO_4$ (x = 0, 1, 3, and 5%) NRDs.

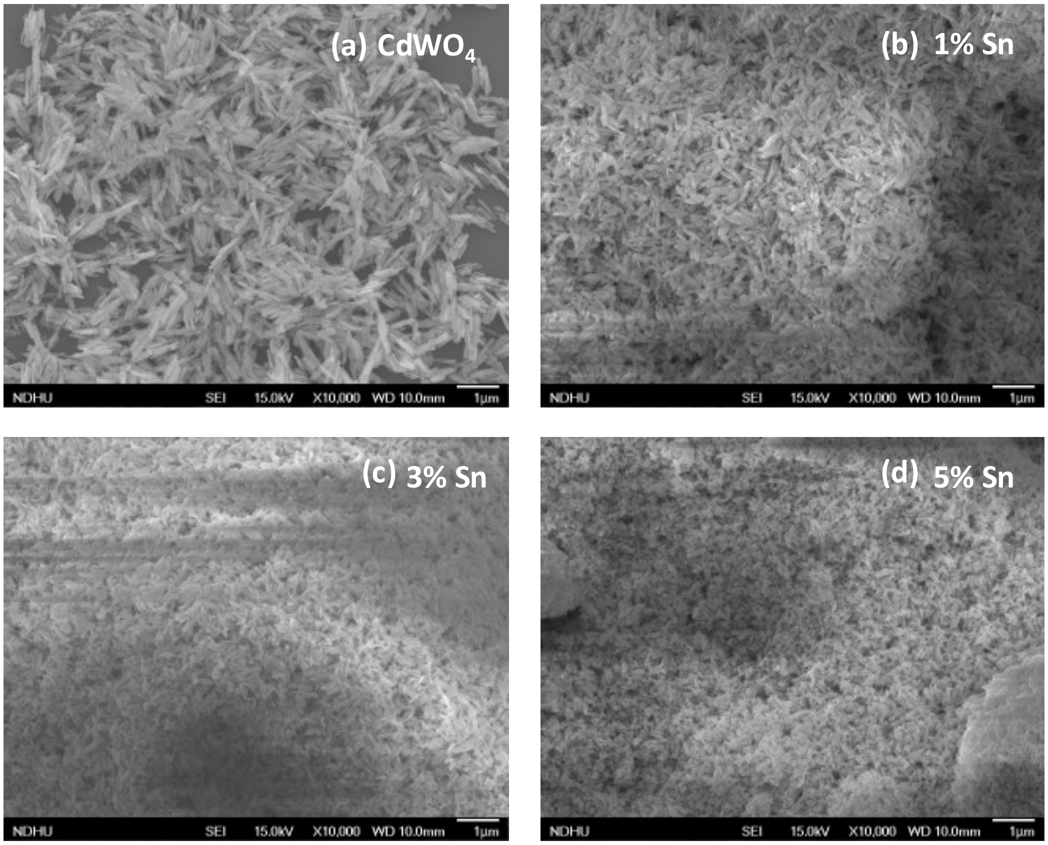

Figure S2 (a)-(d) shows the histogram distribution in the length of $Cd_{1-x}Sn_xWO_4$ (x = 0, 1, 3, and 5 %) NRDs.

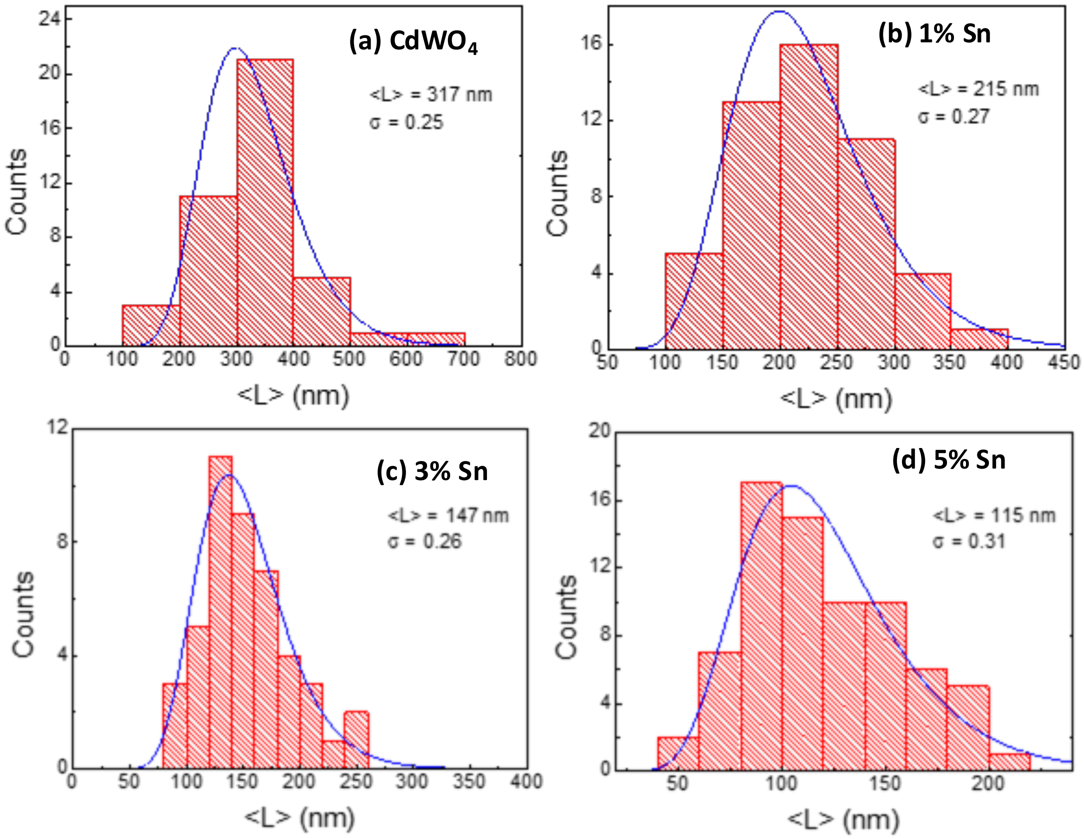

Figure S3 The plot of mean length and diameter v/s Sn concentration estimated from high magnification FE-SEM images.

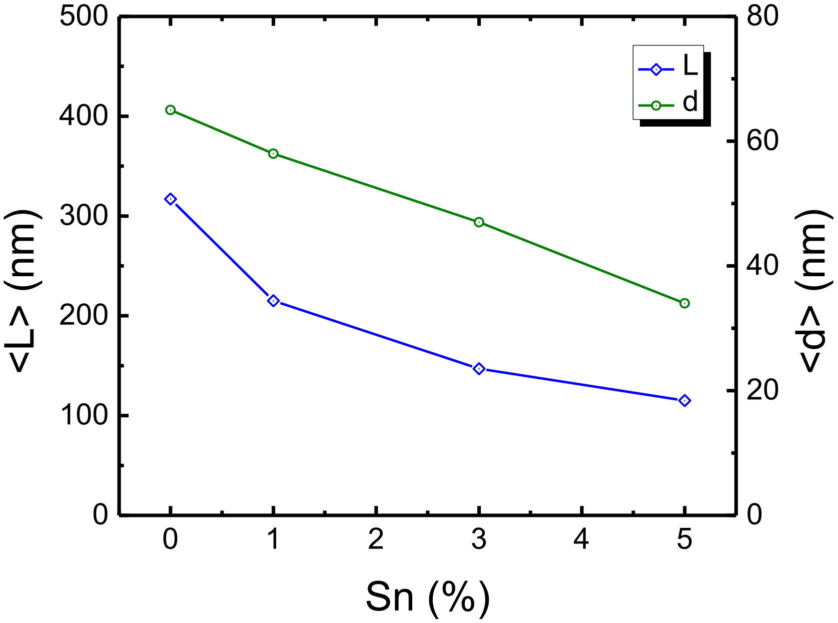

Figure S4 (a)-(d) shows the EDS image of $Cd_{1-x}Sn_xWO_4$ (x = 0, 1, 3, and 5 %) NRDs, respectively.

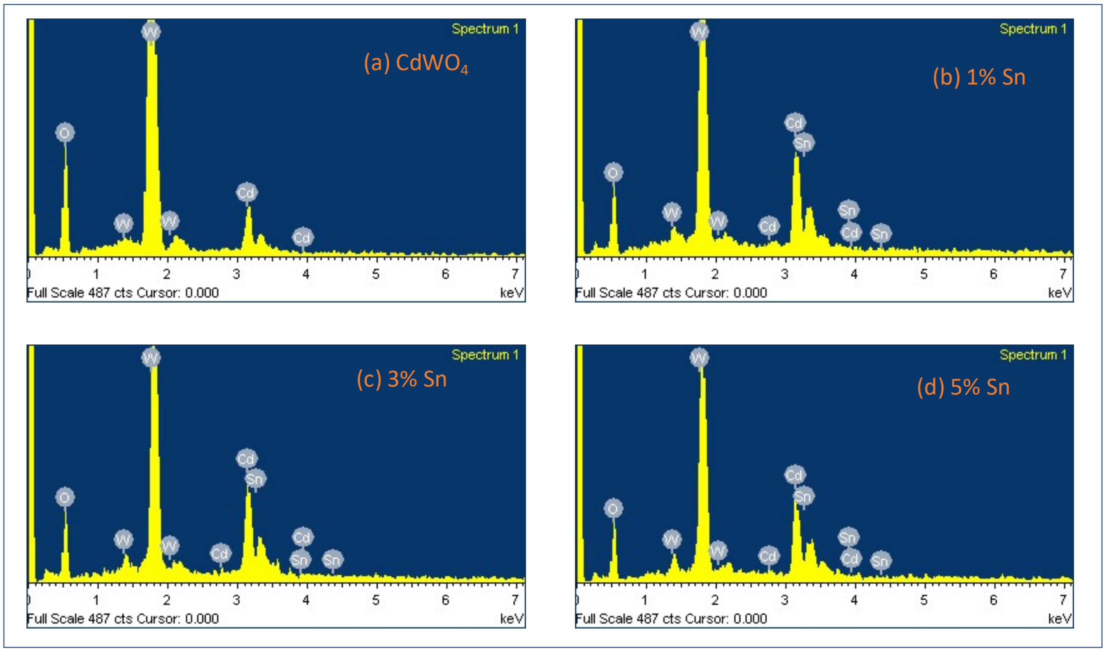

Figures S5 The mean value of d-spacing obtained from FE-TEM images of Sn-doped $CdWO_4$ NRDs.

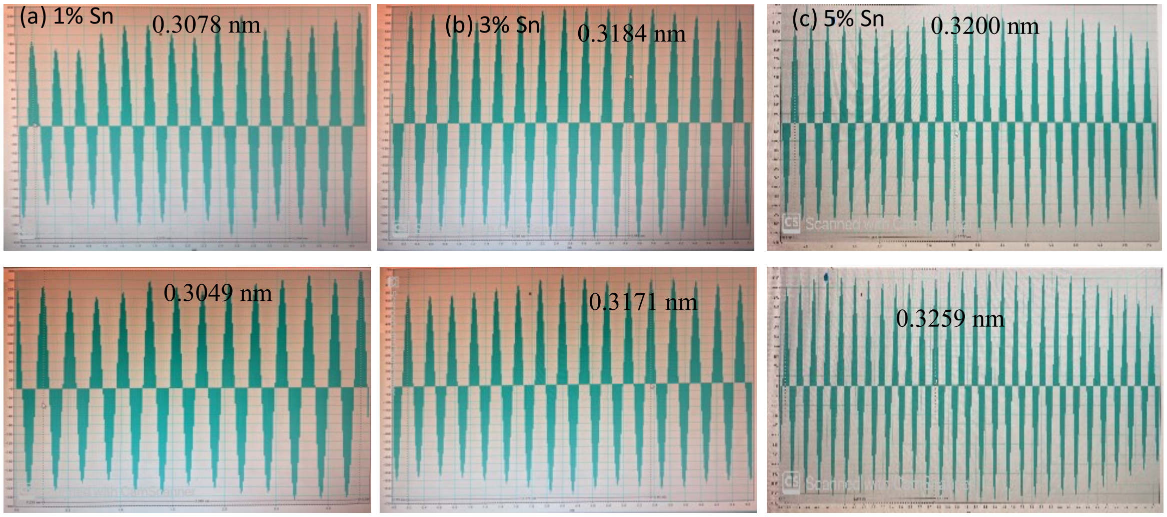

Figure S6 (a)-(d) Maximum intensity XRD peak (-1 1 1) along with Gaussian fit for $CdWO_4$ and 1, 3, 5 % Sn-doped $CdWO_4$ NRDs (Black line represents measured data and the red line indicates Gauss fit of a maximum intense peak.

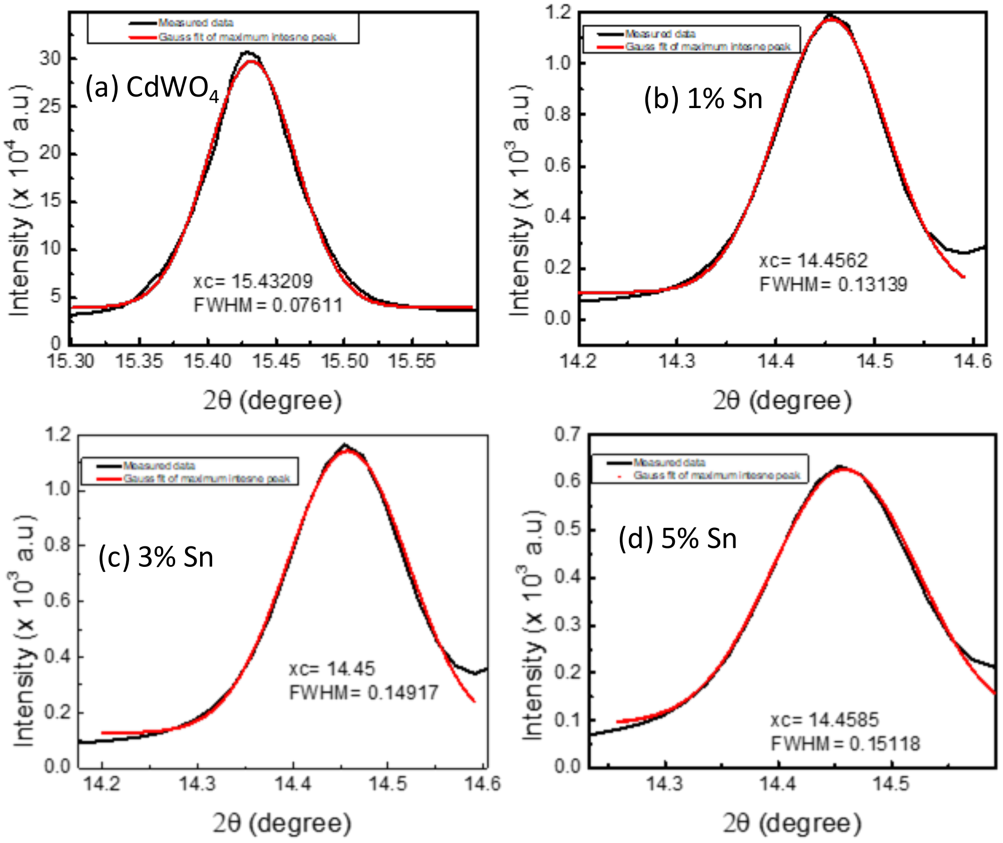

Figure S7 Williamson-Hall plot obtained from the PXRD diffractogram of $CdWO_4$ and 1, 3, 5 % Sn-doped $CdWO_4$ NRDs.

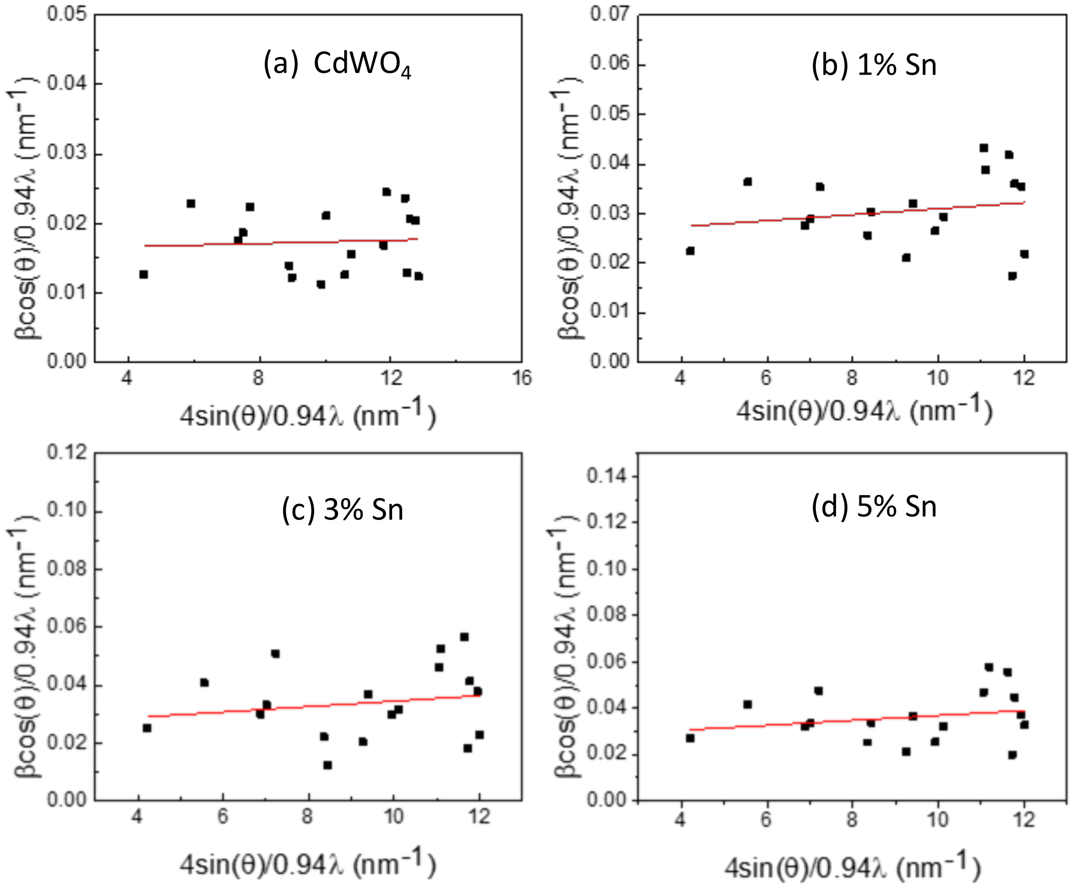

Figure S8 shows Temperature-dependent photoluminescence spectra of undoped and Sn-doped $CdWO_4$ for emission peak at the direct bandgap at 15 to 300 K.

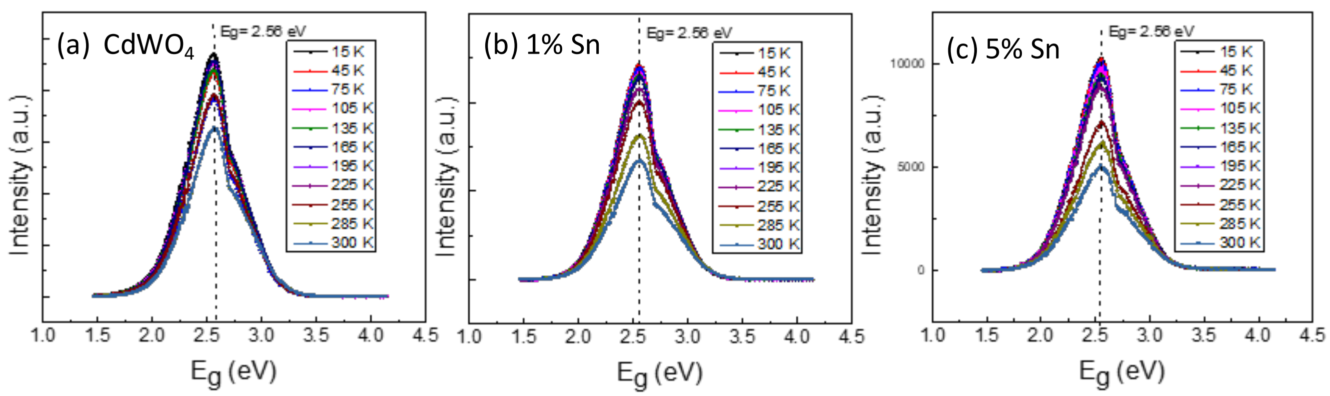

Table S1 shows the mean diameter and mean length obtained from the high magnification SEM images of $Cd_{1-x}Sn_xWO_4$ (x = 0, 1, 3, and 5 %) NRDs.

Sample	SEM			
	$\langle d_{SEM} \rangle$ (nm)	σ_d (nm)	$\langle L \rangle$ (nm)	σ_L (nm)
$CdWO_4$	65	0.25	317	0.25
1%	58	0.42	215	0.27
3%	47	0.25	147	0.26
5%	34	0.21	115	0.31

Table S2 Summarized the best Rietveld refined fitting parameters.

Element		$CdWO_4$	1% Sn	3% Sn	5% Sn
	a	5.0298	5.0279	5.0270	5.0249
	b	5.8610	5.8644	5.8683	5.8696
	c	5.0753	5.0778	5.0794	5.0792
	β	91.4923	91.4927	91.4842	91.4669
	V	149.570	149.673	149.786	149.812
Occupancy	Cd	0.998	0.995	0.982	0.961
	W	1.000	1.000	1.000	1.000
	O(1)	0.983	1.000	0.980	0.990
	O(2)	0.989	1.000	0.980	0.960

Table S3 Summarization of the Sn-doped $CdWO_4$ vibration modes.

Phonon symmetry	ν (cm^{-1})			
	$CdWO_4$	1%	3%	25%
A_g	99	99	99	99
B_g	117	117	117	117
B_g	134	134	134	134
B_g	148	148	148	148
A_g	178	177	176	176
A_g	230	229	228	228
B_g	248	248	248	248
B_g	269	269	268	268
A_g	307	307	307	307
B_g	352	352	352	352
A_g^*	387	387	387	387
B_g	516	516	516	516
A_g^*	548	547	547	546
B_g^*	687	687	687	687
A_g^*	707	707	707	707-
B_g^*	772	772	772	772
A_g^*	898	898	897	896

Table S4 Summarized the best-fitted parameters of Figure 7a-c using equations (3)-(4).

Sample	E_1 (meV)	E_2 (meV)	A	B	IQE (%)
$CdWO_4$	145.72	-	154	-	64.7
1%	192.73	5.1	1201	0.22	55.9
5%	108.8	4.3	1321	0.18	48.9
